# Morphological changes in rat rectus abdominis muscle induced by diabetes and pregnancy

**DOI:** 10.1590/1414-431X20177035

**Published:** 2018-03-01

**Authors:** G. Vesentini, G. Marini, F. Piculo, D.C. Damasceno, S.M.M. Matheus, S.L. Felisbino, I.M.P. Calderon, A. Hijaz, A.M.P. Barbosa, M.V.C. Rudge

**Affiliations:** 1 Departamento de Ginecologia e Obstetrícia, Faculdade de Medicina de Botucatu, Universidade Estadual Paulista, SP, Brasil; 2 Departamento de Ciências da Saúde, Universidade do Sagrado Coração, SP, Brasil; 3 Departamento de Anatomia, Instituto de Biociências de Botucatu, Universidade Estadual Paulista, SP, Brasil; 4 Departamento de Morfologia, Instituto de Biociências de Botucatu, Universidade Estadual Paulista, SP, Brasil; 5 Department of Urology, Urology Institute, University Hospitals Case Medical Center, Cleveland, OH, USA; 6 Departamento de Fisioterapia e Terapia Ocupacional, Universidade Estadual Paulista, SP, Brasil

**Keywords:** Diabetes, Pregnancy, Collagen, Skeletal muscle, Rats

## Abstract

The urethral muscle of diabetic pregnant rats is affected by long-term mild diabetes and short-term severe diabetes, which plays a crucial role in the pathogenesis of pelvic floor disorders. We hypothesized that muscles outside the pelvis are subject to similar changes. The current study aimed at analyzing the effects of long-term mild and short-term severe diabetes on the structure and ultrastructure of fiber muscles and collagen in rats' rectus abdominis (RA) muscle. Therefore, the RA muscle of virgin, pregnant, long-term mild diabetic, short-term severe diabetic, long-term mild diabetic pregnant and short-term severe diabetic pregnant 3-month-old Wistar rats were collected. The structure was analyzed by picrosirius red staining, immunohistochemistry for fast and slow muscle fibers and transmission electron microscopy. We investigated two levels of STZ- induced diabetes: long-term mild diabetes (blood glucose level: 120–200 mg/dL) and short-term severe diabetes (blood glucose level >300 mg/dL). Long-term mild diabetic pregnant and short-term severe diabetic pregnant rats had decreased fast fibers and increased slow fibers, disrupted areas of sarcomere, intermyofibrillar mitochondria and myelin figures in the RA muscle. Both groups enabled us to analyze the specific influence of pregnancy, separately from diabetes. The current study demonstrated that diabetes and pregnancy induced intramuscular transformation and reorganization of RA muscle with a switch of fiber type adjusting their architecture according to intensity and duration of hyperglycemic insult within pregnancy.

## Introduction

Screening strategy, diagnosis, and treatment of gestational diabetes by American Diabetes Association are unable to prevent the high prevalence of urinary incontinence (UI) in women with gestational diabetes mellitus (GDM) (1,2). Such findings motivated our hypothesis that hyperglycemia in pregnancy negatively impacts the pelvic floor muscle function, which plays an important role in the pathogenesis of pelvic floor disorders (1,2). Diabetes is a major factor in total economic costs, i.e. US$322 billion in 2012 (3). In addition, the direct management of UI leads to a cost of US$19.5 billion, which will continue to advance (4). It is possible to infer that GDM and UI would increase women health costs and represent a substantial economic burden for not only individual patients, but also health care systems. Diabetes-related chronic hyperglycemia has been associated with damage in different tissues, including lower urinary tract and striated skeletal muscle (5). The term "diabetic myopathy" refers to function, metabolic and structural changes that are induced by diabetes mellitus (DM) in skeletal muscle (6,7).

A high prevalence of pelvic floor muscle (PFM) dysfunction and decreased vaginal squeeze pressure two years after cesarean delivery in women with GDM (1) inspired us to conduct translational studies using diabetic pregnant rats (8–10), since ethical issues for collection of human tissue might be impeding; therefore, animal models become valuable for studying UI pathophysiology (11). Previous studies have demonstrated that intramuscular changes occur in urethral striated muscles of streptozotocin (STZ)-induced pregnant rats using two different models: short-term severe diabetes (blood glucose level >300 mg/dL) (8,10) and long-term mild diabetes (blood glucose level between from 120 to 300 mg/dL) (9,10). The urethral striated muscle was thin, atrophic, disorganized and associated with decreased expression of fast fibers, as well as increased expression of slow fibers. These findings suggested that PFM dysfunction detected in diabetic pregnant women might reflect changes in urethral striated muscle (1,8–10). Furthermore, research groups have reported a co-contraction mechanism between the abdominal wall muscles and PFM in women (12,13) and the physiological participation of the abdominal wall during voiding in rats (14), showing an important interaction between abdominal wall muscles and PFM both in rats and humans. Thus, morphology and architecture of the rat’s abdominal muscles represent a valid model of the human abdominal wall musculature (15).

Some studies have already shown changes in the skeletal muscle as a result of DM (16,17). In order to assess such changes, these studies have used several types of diabetes induction, hyperglycemic levels and diabetes exposure times. The understanding of the impact of hyperglycemia intensity and duration within pregnancy on skeletal muscle morphology is still not clear, limiting the offer of means for prevention.

Due to the many considerable ethical constraints in assessing human pelvic floor tissue to perform bench-to-bed approach, we used a rat model to observe whether changes are specific to urethral striated skeletal muscle or other muscles.

We hypothesized that the levels and/or duration of hyperglycemic insult associated with pregnancy would lead to similar changes in rectus abdominis (RA) muscle and extracellular matrix content, specifically collagen. The current study aimed to analyze the effects of intensity and duration of hyperglycemic insult within pregnancy on the structure and ultrastructure of fiber muscles and collagen of RA muscle in rats.

## Material and Methods

### Animals

**Figure 1.**
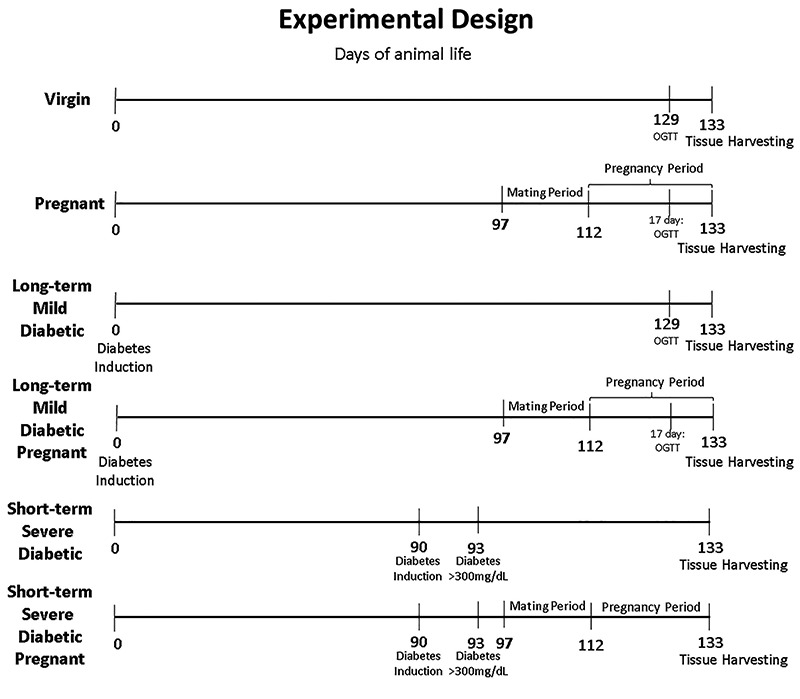
Experimental design. OGTT: oral glucose tolerance test.

Institutional Animal Care and Use Committee in the Faculdade de Medicina de Botucatu (Universidade Estadual Paulista) approved the current study (protocol #1003–2013). Male and female Wistar rats (12–13 weeks, 250–300 g) were obtained from Multidisciplinary Center for Biological Investigation (Campinas, SP, Brazil). Animals were kept at the Gynecology and Obstetrics Laboratory of Experimental Research in polypropylene cages under controlled conditions at room temperature 22±2°C, relative humidity of 55±5%, 12/12 light/dark cycle and fed *ad libitum* (Figure 1).

Seventy-eight rats were randomly distributed into 6 groups with 13 animals each: virgin, pregnant, long-term mild diabetic, short-term severe diabetic, long-term mild diabetic pregnant, and short-term severe diabetic pregnant. All animals were euthanized at the end of the experiment. Exposure time, blood glucose levels and pregnancy determined the aforementioned groups.

#### Long-term mild diabetic rats

On the first day of life, female newborns received STZ (Sigma Chemical Co., USA), diluted in citrate buffer (0.1 M; pH 4.5) at a dose of 100 mg/kg, using a subcutaneous route according to Iessi et al. (18). The STZ-treated rats that presented blood glucose levels higher than 400 mg/dL at the 5th day of life were included in the long-term mild diabetic group. Blood glucose concentrations were measured using a One-Touch Ultra glucometer (LifeScan, Johnson and Johnson¯, USA), and values are reported as mg/dL (18).

#### Short-term severe diabetic rats

Diabetes was induced in adult female rats (90 days of age) using STZ injection (Sigma Chemical Co.). The STZ was administered intraperitoneally at doses of 50 mg/kg to induce a permanent and severe diabetic state. At 72 h after STZ injection, blood samples were obtained to confirm severe diabetic levels, and rats that presented blood glucose levels superior to 300 mg/dL were included in the short-term severe diabetic groups (19).

#### Virgin and pregnant rats

Both non-diabetic groups received citrate buffer at a similar volume and period according to the long-term mild and short-term severe diabetic groups. These rats presented glycemia lower than 120 mg/dL and were included in either virgin or pregnant groups.

### Mating period

Adult female rats were kept overnight with non-diabetic adult male rats. To verify if mating had occurred, female rats were checked early the following morning for spermatozoa in vaginal smear. If positive, it was considered day zero of pregnancy.

### Tissue harvesting

On the 21st day of pregnancy, pregnant and non-pregnant rats were euthanized by sodium thiopental injection (Thiopentax¯, Brazil 80 mg/kg dose). Fragments of the lower third of the RA muscle were immediately obtained. For structural examination, samples were rolled in talcum powder, frozen in liquid nitrogen and stored at -80°C. For ultrastructure analyses, samples were immersed in Karnovsky's fixative.

### Structural examination

The frozen muscle specimens were cut into 10-μm thick cross-sections using a cryostat (Leica CM 1800, Germany). The cross-sections were fixed on microscope glass slides in cold acetone for 10 min, stained with hematoxylin and eosin (H&E) or picrosirius red, and processed for immunohistochemical analysis (n=10 samples/group). The slides were examined using a light microscopy and subsequently photographed (DMR, Leica¯ coupled with digital camera CCD-IRIS/RGB, Sony¯, Germany).

H&E-stained slides were used to observe the general morphology. Slides stained with picrosirius red were analyzed with the color-segmentation method to determine the red- (collagen) and yellow (muscle fiber)-stained tissue in the same section. For analysis, 10 sections/animal were selected for morphometric analysis of collagen area and muscle area (20× magnification) using Image Pro Plus 7.0 image analysis software (Media Cybernetics, at Case Western Reserve University, USA). This software can distinguish and accurately measure fields stained with different colors by counting and converting the pixels in the image into a value for the area (μm^2^).

#### Immunohistochemistry

Immunohistochemical analysis was used to stain fast and slow-type skeletal muscle fibers. Frozen sections were hydrated for 10 min; endogenous peroxidase was blocked by using H_2_O_2_ (3%) in a methanol solution (97%). After washing, sections were incubated with 3% fetal bovine serum at 37°C. The sections were incubated overnight at 4°C with antibodies against WB-myosin heavy chain, fast (WB-MHCf, Novocastra, 1:120) or WB-MHC slow (WB-MHCs, Novocastra, 1:180). Then, samples were washed three times with 0.01 M PBS. Dako LSAB¯ System-HRP Detection System (Denmark) was utilized for further incubation at room temperature, and slides were washed three times with 0.01 M PBS. For staining, the samples were incubated with 3.3-diaminobenzidine tetrahydrochloride (Sigma-Aldrich) for 5 min, 0.01 M PBS for 10 min and hematoxylin for 10 min. The localization of fast and slow fibers was done using a light microscopy (DMR, Leica¯ coupled with a CCD-IRIS/RGB digital camera, Sony¯), and the fiber type area was quantified using the image processing and analysis Java software ImageJ (National Institutes of Health, USA). This software measures and counts each fiber area after manually defining and converting the pixels in the image into a value for the area (μm^2^). The area was determined after measuring ∼200 muscle fibers from each animal. In addition, the fiber type number in 4 sections/animal was counted using the ImageJ software.

#### Ultrastructural analyses

The samples (n=3 samples/group) were immersed in Karnovsky's fixative for 24 h prior to post-fixation in osmium tetroxide. Subsequently, specimens were embedded in epoxy resin. The sections were obtained using an ultramicrotome at a longitudinal orientation, and stained sections were examined using transmission electron microscopy (Philips CM 100, The Netherlands).

### Statistical analysis

Results are reported as means±SD. Comparisons between muscles and conditions were performed by repeated measures 2-way analysis of variance to avoid over influence from a single group (virgin, pregnant, long-term mild diabetes, short-term severe diabetes, long-term mild diabetes pregnant and short-term severe diabetes pregnant). Comparisons between individual groups were made using multiple comparisons with Student's *t*-test, as appropriate. Poisson distribution was performed when data presented no homogeneous distribution, such as fiber type number. Pearson's correlation test was used to assess the correlation between variables (offspring number, litter weight, weight gain during pregnancy, collagen area, muscle area, slow fiber number and area, fast fiber number and area, and blood glucose levels) in pregnant groups. The significance level was set at P<0.05. All statistical analyses were performed using SAS software version 9.2. (Statistical Analysis System Institute Inc., USA).

## Results

Results indicated that long-term mild diabetic pregnant and short-term severe diabetic pregnant had decreased fast fibers and increased slow fibers in RA muscle. The four control groups, i.e., virgin, pregnant, long-term mild diabetic and short-term severe diabetic, allowed us to analyze the influence of pregnancy, separated from diabetes.

Fibers presented no morphological differences using color segmentation analysis; however, further characterization using picrosirius red, fast and slow immunostaining and ultrastructural analysis was effective to identify such differences.

### General morphologic characteristics

#### Virgin group

**Figure 2.**
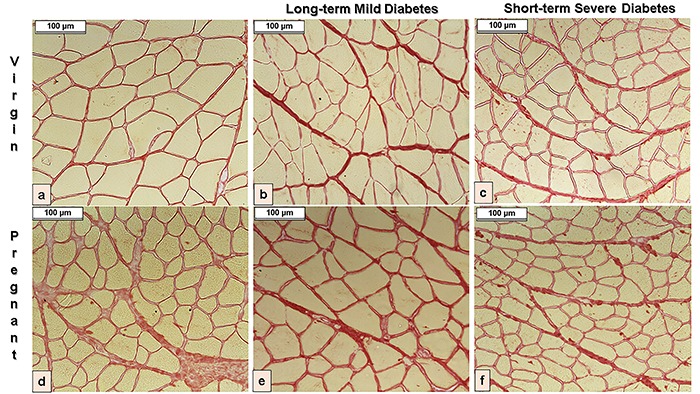
Photomicrographs of rat rectus abdominis after picrosirius red staining of striated muscle (yellow) and collagen (red). Virgin (*a*), long-term mild diabetic (*b*), short-term severe diabetic (*c*), pregnant (*d*), long-term mild diabetic pregnant (*e*), and short-term severe diabetic pregnant (*f)*. Magnification 20×.

**Figure 3.**
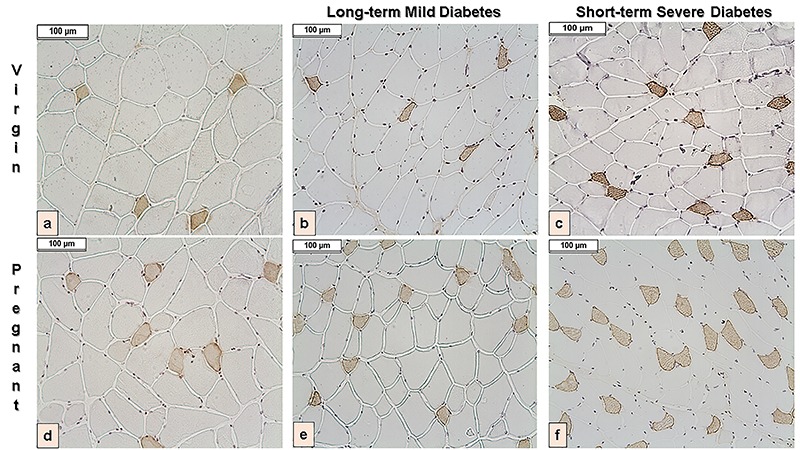
Immunohistochemistry images of slow fibers in a transverse section of rat rectus abdominis. Virgin (*a*), long-term mild diabetic (*b*), short-term severe diabetic (*c*), Pregnant (*d*), long-term mild diabetic pregnant (*e*), and short-term severe diabetic pregnant (f). Magnification 20×.

**Figure 4.**
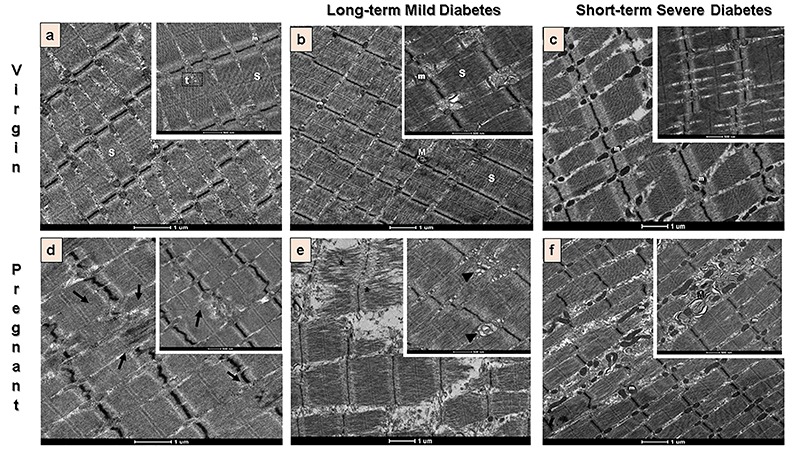
Electron micrographs of rat rectus abdominis muscle fibers from virgin (*a*), long-term mild diabetic (*b*), short-term severe diabetic (*c*), pregnant (*d*), long-term mild diabetic pregnant (*e*), and short-term severe diabetic pregnant (*f*) animals. The micrographs show mitochondria (m), myelin figures (M), well-organized myofibrils forming intact sarcomeres (S) and a system formed by sarcoplasmic reticulum and t tubule-triads (t), areas with disorganized Z lines and thinning sarcomeres (arrows), swollen sarcoplasmic reticulum and dilated T tubes (arrowheads) and areas with disrupted sarcomeres (*). Inset scale bar: 500 nm.

The RA of healthy adult rat females was comprised of different size fibers with polygonal and peripheral myonuclei. By immunolocalization, a high proportion of fast fibers were detected, which were also generally larger than slow fibers (Figure 2a). Each fiber was surrounded by collagen, and a thick layer of connective tissue involving a small group of fibers (Figure 3a). In the ultrastructural analysis, the RA showed well-organized myofibrils forming intact sarcomeres and organized triads, which are a system formed by sarcoplasmic reticulum and t tubules, with morphological traits associated with different muscle fiber types and a normal distribution of intermyofibrillar mitochondria (Figure 4a).

#### Pregnant group

**Figure 5.**
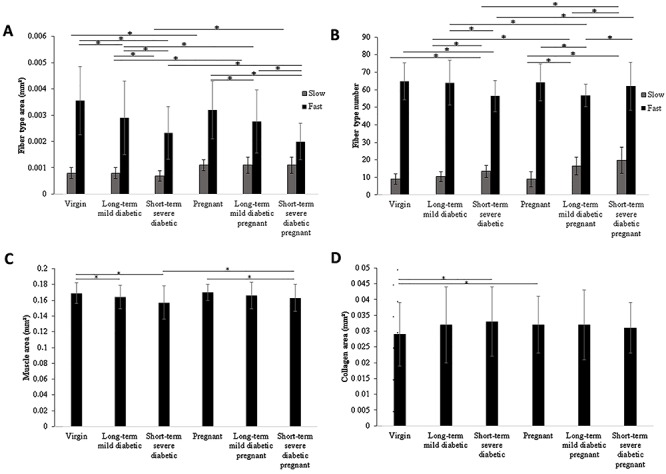
Comparison of fiber type area (*t*-test) (*A*), fiber type number (Poisson test) (*B*), muscle area (*t*-test) (*C*), and collagen area (*t*-test) (*D)*. Data are reported as means±SD. *P<0.05.

The RA of this group were morphologically different from those obtained in the virgin group, with an increased number of slow fibers and increased collagen area (Figures 2d, 3d, 5a and 5d). Disorganized Z lines, thinned sarcomeres, and a usual form and quantity distribution of intermyofibrillar mitochondria were observed (Figure 4d).

#### Long-term mild diabetic group

This group showed a decrease in muscle and fast fiber area compared to the virgin group (Figures 2b, 3b, 5a and 5c). There was no difference in the collagen area (Figures 3b and 5d). In the ultrastructure analysis, the long-term mild diabetic group presented well-organized myofibrils and myelin figures associated with degenerated organelles (Figure 4b).

#### Short-term severe diabetic group

This group presented a significantly decreased number of fast fibers and a reduced slow fiber area compared to virgin and long-term mild diabetic groups. In addition, the number of slow fibers was higher than virgin and long-term mild diabetic groups. However, the fast fiber area decreased compared to virgin and long-term mild diabetic groups (Figures 2c, 5a and 5b). The muscle area was decreased compared to virgin and short-term severe diabetic pregnant groups. The collagen area was increased compared to virgin group (Figures 3c and 5d). The ultrastructural analysis revealed numerous subsarcolemmal, intermyofibrillar mitochondria and striated muscle cells thinning in the muscle fibers (Figure 4c).

#### Long-term mild diabetic pregnant group

This group had a decreased number of fast fibers and area compared to long-term mild diabetic and pregnant groups. Moreover, the slow fibers number and area were higher than long-term mild diabetic group, whereas a higher number of slow fibers was observed compared to the pregnant group (Figures 2e, 5a and 5b). There was no difference regarding the collagen and muscle area in the two-way analysis between the groups (Figures 3e, 5c and 5d). Ultrastructural analysis showed swollen sarcoplasmic reticulum, dilated t tubes and areas with sarcomere disruption (Figure 4e).

#### Short-term severe diabetic pregnant group

The quantitative analysis showed an increased number of fast fibers and a decrease in fast fiber area compared to short-term severe diabetic pregnant and long-term mild diabetic pregnant groups. The slow fiber number and area were higher than those in short-term severe diabetic group. In addition, a higher number of slow fibers compared to pregnant and long-term mild diabetic pregnant groups were observed (Figures 2f, 5a and 5b). The muscle area was greater compared to the short-term severe diabetic group and smaller compared to the pregnant group. In the collagen area, no difference was found between groups (Figures 3f and 5d). Ultrastructural analysis showed an increase in intermyofibrillar mitochondria and myelin figures (Figure 4f).

**Figure 6.**
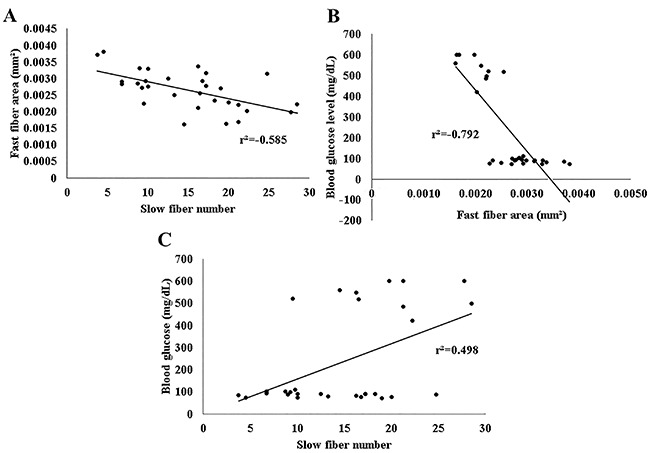
Pearson’s correlation for fast fiber area and slow fiber number (*A*), fast fiber area and blood glucose level (*B*), and slow fiber number and blood glucose level (*C*).

Pearson's correlation analysis showed a significant negative correlation between fast fiber area and slow fiber number (r=-0.585, P=0.0007; Figure 6A). Blood glucose level was negatively correlated with fast fiber area (r=-0.792, P=<0.0001; Figure 6B), however it was positively correlated with slow fiber number (r=0.498, P=<0.005; Figure 6C).

## Discussion

Many studies have contributed to the understanding of the additive or synergistic effects of muscle function in diabetic women or rats. Also, many hypotheses have been proposed based on scientific evidence obtained from translational studies to explain the relationship between DM and muscle dysfunction (1,2,8–10). However, the current study is the first investigation of structural and ultrastructural alterations of RA muscles of STZ-induced diabetic pregnant rats.

Current data demonstrated that pregnancy and DM induce adaptations in rat RA muscle by systematically adjusting architectural design in each fiber type. An increase in fiber type area and number was detected in the integrated morphological analysis of rat RA in long-term mild or short-term severe diabetic pregnant rats. Our results showed an increased number of slow fibers in both diabetic pregnant models. Although a significant decreased in fast fiber number occurred in long-term mild diabetic, fast fiber number increased in short-term severe diabetic. Therefore, we were somewhat surprised to find a negative change of fiber types in the long-term mild diabetic pregnant group compared to short-term. Possibly, a long-term mild hyperglycemic insult can have more severe detrimental impact in architectural parameters than short-term severe hyperglycemic insult. Such finding suggested a potential influence of the duration of hyperglycemic insult. Moreover, a switch from fast to slow fibers supposedly represents an adaptive response to hyperglycemic status on muscle more related to hyperglycemic duration (10).

The pathophysiological cycle of diabetic myopathy was established after Pearson correlation analysis (Figure 6). High blood glucose levels directly caused a decrease in fast fiber area during pregnancy both in diabetic and non-diabetic rats; such persistent decrease in fast fiber area led to an increase in slow fiber number that was associated with high blood glucose concentrations during diabetic pregnancy. Skeletal muscle nutrient-related atrophy, such as that observed in diabetes, reflects different intracellular pathways that are associated with protein degradation abnormalities in fast fibers (20). Increased slow fibers may result from higher glucose handling capacity from this type of fiber (21). Similar changes in the normal architecture of muscles subjected to a hyperglycemic environment were observed in the current study, which are consistent with previous literature (7–10,16,17).

Diabetes is associated with increased collagen, which plays a crucial role in muscle regeneration (22). In the current study, there was no change in the collagen area of the RA muscle in both diabetic pregnant groups, contrary to changes observed in collagen content of other tissues using the same diabetic model. Previously, we showed an increased collagen area in urethral striated muscle of the long-term mild diabetic pregnant group (8–10). The reason for the discrepancy between different tissue compositions remains elusive. The striated urethral muscle closely contacts urothelium and smooth muscle. Conversely, the skeletal muscle extracellular matrix (ECM) surrounds the muscle fibers. Krause et al. (23) demonstrated that increased expression of collagen is important for maintaining muscle integrity. In contrast, excessive collagen levels are pathological, leading to fibrosis and affecting normal regenerative process, with impaired infiltration of macrophages and muscle satellite cells into damaged tissue areas. These changes might be associated with a negative impact on muscle function, such as fibrotic muscles, leading to muscle atrophy (24). The current results showed that combined diabetes and pregnancy, two important factors for increased collagen, were not enough to develop skeletal muscle fibrosis in RA.

Previous reports (25,26) showed an increased slow fiber area in pregnant groups compared to virgin groups, which indicates that pregnancy was the major factor. Individually, the muscle fibers show differences in contraction velocity, oxidation, capillarity, and number and size of the mitochondria (27). The relative balance of fiber biosynthesis versus fiber degradation is important for maintaining muscle mass (20). However, the mechanisms implicated in this balance are affected by pregnancy, potentially explaining the differences observed in muscle area. This might reflect the influence of estrogen, which contributes to endogenous fiber type differences, as content and distribution of estrogen receptors in skeletal muscle are more highly expressed in slow fibers than in fast fibers (28). The increase in collagen content of the pregnant group corresponds to the impact that pregnancy has on the skeletal muscle architecture, as the ECM is a passive structure with capacity of sustaining load. ECM collagen stabilizes the elongated sarcomeres and protects the muscle fibers from excessive stretching of the abdominal wall during pregnancy and parturition, supplying an elastic element that limits fiber tension (26,29). Ultrastructure images indicated stretched areas with disorganized Z lines and thinned sarcomeres (Figure 4d), as a result of pregnancy.

The interpretation of the results obtained using the STZ-induced diabetic model should be cautiously made because this diabetic induction occurred before pregnancy in contrast with GDM that is developed during pregnancy. Future animal studies should use alternative models, considering the current knowledge of the effects of diabetes on skeletal muscle. Even though there are inherent limitations in the use of a quadrupedal animal model, the abdominal muscles of rats and humans have been previously described as similar concerning architectural and morphological properties (15). Due to the differences in posture of rats and humans and to the pre-pregnancy diabetes induction, such model is valuable to establish structural adaptations in rat RA muscle. The current study represents a significant step towards future studies examining GDM effects in RA muscle during a cesarean section.

In conclusion, our findings demonstrated an important adaptation to excessive mechanical tension, showing intramuscular transformation and reorganization in fiber types of diabetic pregnant rat RA. The adjustment of muscle architecture according to the metabolic or mechanical environment could contribute to muscle dysfunction. These results confirm RA muscle fiber adaptation in pregnant rats with short-term severe diabetes, as well as pregnant rats with long-term mild diabetes showing that muscles outside the pelvis are subjected to similar structural changes related to diabetic myopathy (8–10). Understanding the pathophysiological mechanisms that underlie diabetic myopathy, as a systemic disease, is relevant to the development of appropriate and successful long-term therapeutic strategies to improve quality of life.
